# Breast self-examination and death from breast cancer: a meta-analysis

**DOI:** 10.1038/sj.bjc.6600847

**Published:** 2003-04-01

**Authors:** A K Hackshaw, E A Paul

**Affiliations:** 1 Barts & The London School of Medicine & Dentistry, Wolfson Institute of Environmental & Preventive Medicine, Queen Mary, University of London, Charterhouse Square, London EC1M 6BQ, UK

**Keywords:** breast self-examination, breast cancer, mortality, meta-analysis

## Abstract

Breast self-examination (BSE) is widely recommended for breast cancer prevention. Following recent controversy over the efficacy of mammography, it may be seen as an alternative. We present a meta-analysis of the effect of regular BSE on breast cancer mortality. From a search of the medical literature, 20 observational studies and three clinical trials were identified that reported on breast cancer death rates or rates of advanced breast cancer (a marker of death) according to BSE practice. A lower risk of mortality or advanced breast cancer was only found in studies of women with breast cancer who reported practising BSE before diagnosis (mortality: pooled relative risk 0.64, 95% CI 0.56–0.73; advanced cancer, pooled relative risk 0.60, 95% CI 0.46–0.80). The results are probably due to bias and confounding. There was no difference in death rate in studies on women who detected their cancer during an examination (pooled relative risk 0.90, 95% CI 0.72–1.12). None of the trials of BSE training (in which most women reported practising it regularly) showed lower mortality in the BSE group (pooled relative risk 1.01, 95% CI 0.92–1.12). They did show that BSE is associated with considerably more women seeking medical advice and having biopsies. Regular BSE is not an effective method of reducing breast cancer mortality.

For many years, women have been taught methods of breast self-examination (BSE) and it is recommended that they practise this regularly (Boyle *et al*, 1995; Shapiro *et al*, 1998), usually every month. There is a belief that among women who practise BSE, those who develop breast cancer are more likely to find it at an earlier stage and this is expected to lead to earlier treatment and hence decrease their risk of dying from the disease. Breast self-examination is appealing as a routine screening method because the examination has no financial cost (apart from the initial instruction sessions) and can be conducted in private. Most studies on the effectiveness of BSE have been observational. They suggest that women who practise BSE are more likely to find their breast tumour themselves, that the tumour tends to be smaller and that these women have an increased survival (Hackshaw, 1996; International Agency for Research on Cancer (IARC), 2002). However, survival time as an outcome measure can be misleading because of lead-time bias, in which BSE only identifies cancers at an earlier stage but has no effect on prognosis. Using mortality rates instead of survival time can overcome much of this bias.

Recently, the International Agency for Research on Cancer (2002) published a review on breast-cancer screening that reported the individual results from observational studies of BSE in relation to survival and stage of cancer, and those from randomised trials and cohort studies in relation to mortality. We here, however, present a meta-analysis of BSE and breast-cancer mortality by reviewing the published evidence from both observational studies and randomised trials, including those based on women with advanced breast cancer (used as a marker of death), and pooling the results. We look at three aspects of BSE; women who practise BSE, women who find their cancer during one of their regular examinations, and women who are taught BSE and advised to practise it regularly.

## METHODS

### Data sources and study selection

Studies that reported on rates of death from breast cancer or rates of advanced breast cancer (a marker of death) according to BSE practice were identified from Medline, Embase and Cancerlit (1966–2002), and included in the analysis. Keywords used were ‘breast cancer’ with ‘BSE’ or ‘self-examination’. In some studies, women were classified according to whether they practised BSE regularly or not. In other studies, women were classified according to the method of detecting the cancer: during BSE, by chance (e.g. while washing or dressing), mammography or examination by physician. Below we describe the main common features of the studies, but in the interest of brevity we do not provide further details, since these can be obtained directly from the individual published reports.

We included results on mortality or, as a surrogate for death, advanced breast cancer (defined as stage III or IV, regional or distant). Analyses are presented separately for these two outcomes.

The following types of studies were included in the analyses.

#### Studies on women newly diagnosed with breast cancer

A total of 15 studies were based only on women newly diagnosed with breast cancer (Greenwald *et al*, 1978; Smith *et al*, 1980; Feldman *et al*, 1981; Tamburini *et al*, 1981; Foster and Costanza, 1984; Owen *et al*, 1985; Smith and Burns, 1985; Ogawa *et al*, 1987; Huguley *et al*, 1988; Kuroishi *et al*, 1992; Le Geyte *et al*, 1992; Kurebayashi *et al*, 1994; Auvinen *et al*, 1996; McPherson *et al*, 1997; Koibuchi *et al*, 1998) and they were divided into four groups based on two different measures of outcome and two different measures of exposure.

- Women who reported practising BSE or not and were then followed up for several years (usually about 5 years) to see who later died from breast cancer.
- Women who reported whether they found their cancer during self-examination or by chance and were then followed up for several years to see who later died from breast cancer.
- Women found to have advanced breast cancer at the time of initial diagnosis who reported retrospectively on whether they practised BSE or not.
- Women found to have advanced breast cancer at the time of initial diagnosis who reported retrospectively on whether they had found their cancer during self-examination or by chance (e.g. washing and dressing).

In most studies mammography use was not stated, although it would not have been offered to many women since these studies were based on women diagnosed before the mid-1980s when such screening was not commonplace. In other studies, mammography use was low (2% of cancers detected by mammography, in Feldman *et al*, 1981) or similar between the BSE and non-BSE groups (Smith and Burns, 1985). In one study (Koibuchi *et al*, 1998), all women had a clinical examination as part of a mass-screening programme. A difference between the BSE and non-BSE group was reported with respect to mammography use in one study (18% in the BSE group compared to 7% in the non-BSE group, Huguley *et al*, 1988) and mass screening by clinician examination in another study (37% in the BSE group and 21% in the non-BSE group, Kuroishi *et al*, 1992); analyses were performed both with and without these two studies.

#### Cohort studies of women with and without breast cancer

The two cohort studies were from Finland (Gastrin *et al*, 1994) and the USA (Holmberg *et al*, 1997). In these, breast cancer death rates according to BSE practice were reported in populations of women followed up for over 13 years. In one study (Gastrin *et al*, 1994), mammography was used only as a method of further investigation after a woman found a lump by BSE. In the other study, the follow-up period was until 1972 when mammography was not commonplace.

#### Case–control studies of women with and without breast cancer

There were three case–control studies, two from the USA (Newcomb *et al*, 1991; Muscat and Huncharek, 1992) and one from Canada that was nested within a randomised trial of mammography (Harvey *et al*, 1997). In each study, cases (women who had died from breast cancer or had advanced cancer) and age-matched controls (women without breast cancer) were asked about their past BSE practice. One study also further matched for screening centre and enrolment year.

### Clinical trials

One trial, from the UK, was nonrandomised (UK Trial of Early Detection of Breast Cancer Group, 1999) and two, from China (Thomas *et al*, 1997,^2002^) and Russia (Semiglazov *et al*, 1992,^1996^,^1999^), were randomised. The nonrandomised trial was based on comparing the breast cancer death rates after 16 years follow-up in two centres, in which women aged 45–64 years were invited to attend a BSE session, with the rates in four centres, in which women were not invited for either BSE training or mammography.

The two randomised trials were large. The one from China (Thomas *et al*, 1997,^2002^) was based on randomising 520 factories in Shanghai, in which all women in a particular factory were either given three sessions on how to practise BSE or they were not. In total, there were about 267 000 women aged 30–69 years. Recruitment began in 1989 and interim results were reported after 5 years. The trial in Russia involved two cities (Moscow and St Petersburg (formerly Leningrad)), but only the results from St Petersburg have been published; approximately five (Semiglazov *et al*, 1992), nine (Semiglazov *et al*, 1996) and 13 (Semiglazov *et al*, 1999) years after recruitment began in 1985. This trial included about 120 000 women aged 40–64 years and, similar to the one in China, randomisation was undertaken according to the place of work and BSE was taught during several sessions. Information on the following was also extracted from the reports of the two randomised trials; the number of women who sought medical advice after finding a lump, the number who had a biopsy and the number diagnosed with breast cancer. Mammography screening was not available to women in either trial.

Attendance of the BSE training sessions in the UK trial was low; only 31 and 53% of women in the two centers, respectively, accepted the invitation to be taught BSE. Attendance in the trial from China was high; 98% received baseline instruction and in one cohort with complete information on attendance (representing about half the women in the BSE group in the trial) 84% had attended all three training sessions. The reports from the Russian trial were based on women who had received training in BSE.

### Definition of BSE practice

In the studies based on only women newly diagnosed with breast cancer, the definition of BSE practice varied. It was monthly (Ogawa *et al*, 1987; Auvinen *et al*, 1996), monthly or several times a year (Feldman *et al*, 1981; Tamburini *et al*, 1981; Foster and Costanza, 1984; Koibuchi *et al*, 1998) or at least two (Smith and Burns, 1985) or three (Smith *et al*, 1980) times per year. In several studies, about half or more of the women in the BSE groups had reported that they checked their breasts monthly (Feldman *et al*, 1981; Smith and Burns, 1985; Ogawa *et al*, 1987; Le Geyte *et al*, 1992). In the two cohort studies (Gastrin *et al*, 1994; Holmberg *et al*, 1997) women were classified as BSE practitioners, if they did so monthly. In the Russian trial, 76% of women taught BSE reported practising it at least every 2 months (Semiglazov *et al*, 1999), and in the Chinese trial women practised BSE at least every 4–5 months during the first 4–5 years of the trial and were strongly encouraged to practise it monthly (Thomas *et al*, 2002).

### Statistical analysis

The relative risks (or odds ratio) and 95% confidence intervals (CI) were estimated from the data in each study. They were pooled on a log scale and weighted by the inverse of the variance, with allowance for any heterogeneity (DerSimonian and Laird, 1986).

## RESULTS

Table 1

**Table 1:** Observational studies of women with breast cancer; the number of deaths or advanced cancers and relative risk of dying from breast cancer in women who practise BSE compared to those who do not and in those who found their cancer during an examination

		**Women who practise BSE regularly**		**Women who never or rarely practise BSE**		
**Study (first author), country**	**Age of women (years)**	**No. of breast cancer deaths or advanced cancers**	**No. of women with breast cancer**	**No. of breast cancer deaths or advanced cancers**	**No. of women with breast cancer**	**Relative risk of death or advanced cancer (95% CI)**
*Breast cancer death*						
Foster, 1984, USA	All (20–97)	61	424	108	411	0.55 (0.40–0.75)
	22–49	18	134	15	58	0.52 (0.26–1.03)
	50–97	43	287	92	346	0.56 (0.39–0.81)
Huguley, 1988, USA	All (unspec.)	327	1398	260	681	0.61 (0.52–0.72)
Le Geyte, 1992, UK	15–59	60	226	130	390	0.80 (0.59–1.08)
Kurebayashi, 1994, Japan	All (unspec.)	3	91	10	132	0.44 (0.12–1.58)
Auvinen, 1996, Finland	All (unspec.)	—	246	—	104	0.85 (0.53–1.33)
						
*Advanced breast cancer*						
Smith, 1980, USA	30–80	44	107	24	57	0.98 (0.59–1.61)
Feldman, 1981, USA	All (unspec.)	137	408	256	588	0.77 (0.63–0.95)
Tamburini, 1981, Italy	35–64	34	170	90	330	0.73 (0.49–1.09)
Foster, 1984, USA	All (20–97)	41	422	123	410	0.32 (0.23–0.46)
Smith, 1985, USA	20–54	75	185	67	134	0.81 (0.58–1.13)
Ogawa, 1987, Japan	25–77	3	30	20	116	0.58 (0.17–1.95)
Huguley, 1988, USA	All (unspec.)	225	1396	246	680	0.45 (0.37–0.53)
Kurebayashi, 1994, Japan	All (unspec.)	7	91	18	132	0.56 (0.24–1.35)
Koibuchi, 1998, Japan	All (unspec.)	3	68	18	174	0.43 (0.13–1.45)
						
		**Cancer found during BSE**		**Cancer found by accident**^a^		
*Breast cancer death*						
Greenwald, 1978, USA	All (unspec.)	*16*	*55*	*65*	*182*	*0.81 (0.47–1.41)*
Kuroishi, 1992, Japan	All (unspec.)	—	347	—	1322	0.57 (0.33–0.99)
Auvinen, 1996, Finland	All (unspec.)	—	34	—	104	1.06 (0.88–1.26)
McPherson, 1997, USA	40–49	33	200	70	364	0.86 (0.57–1.30)
						
*Advanced breast cancer*						
Greenwald, 1978, USA	All (unspec.)	11	55	56	182	0.65 (0.34–1.24)
Owen, 1985, USA	All (unspec.)	76	185	539	1168	0.89 (0.70–1.13)
Kuroishi, 1992, Japan	All (unspec.)	28	355	224	1327	0.47 (0.32–0.69)

a In one study (Greenwald *et al*, 1978), 20% of women in this group practised BSE although found their cancer by chance. Italics indicate that the data were estimated from results presented in the paper.

shows results from the observational studies based only on women with breast cancer.

Figure 1

**Figure 1:**
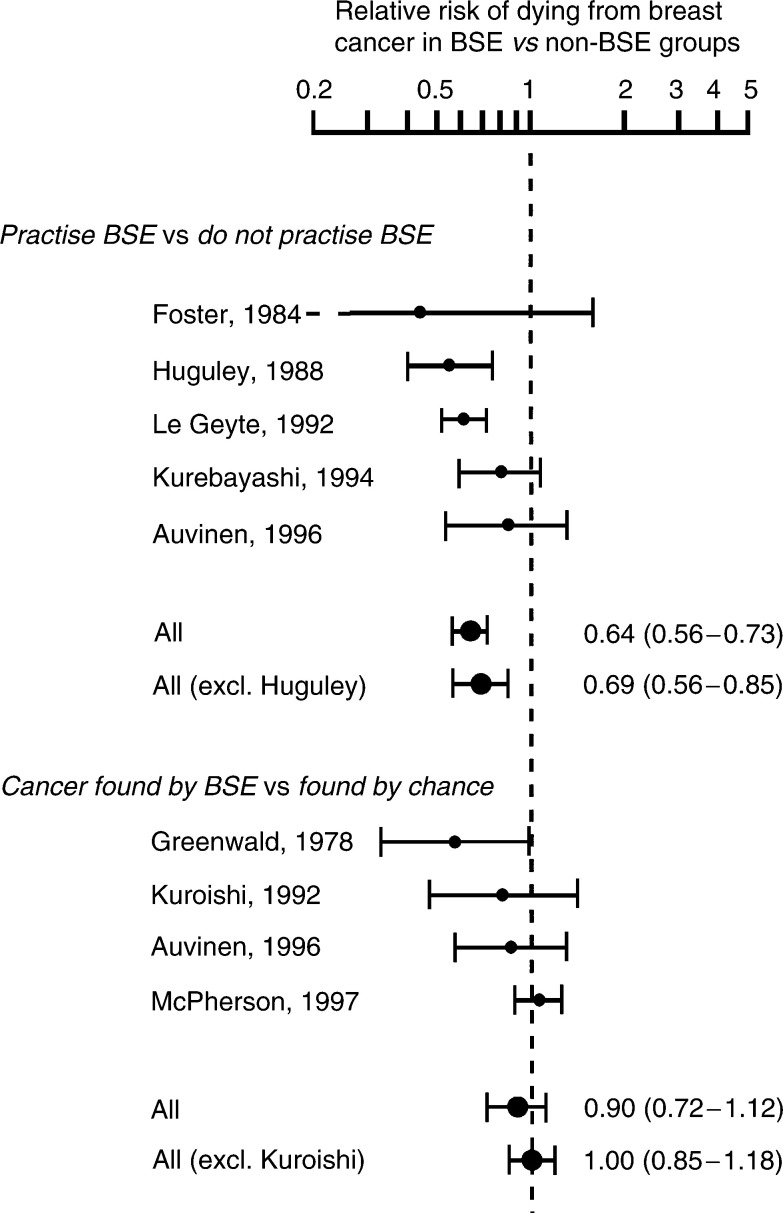
Observational studies of women with breast cancer, comparing the breast cancer death rates between the BSE and non-BSE groups. A test for heterogeneity between the studies yielded a *P*-value of 0.41 for those studies based on women who practise BSE and a *P*-value of 0.26 for those based on finding cancer by BSE.

shows the individual relative risk of death from breast cancer and the pooled relative risks. Overall, there appears to be a statistically significant 36% reduction in the risk of death (relative risk 0.64, 95% CI 0.56–0.73, *P*<0.001) in those who practise BSE. There was no evidence of heterogeneity between the studies (*P*=0.41). If the study in which some women had mammo-graphy (Huguley *et al*, 1988) is excluded, the estimate is not substantially different (relative risk 0.69, 95% CI 0.56–0.85, *P*<0.001). In those women who reported that their cancer was detected during self-examination, there was no evidence of a reduction in the risk of death compared to those who found their cancer by chance (relative risk 0.90, 95% CI 0.72–1.12, *P*=0.34). Again there was no strong evidence of heterogeneity between the results (*P*=0.26). If the study (Kuroishi *et al*, 1992) in which some women had mass screening is excluded, the estimate is not much changed the pooled relative risk is 1.00 (95% CI 0.85–1.18, *P*=0.98).

Figure 2

**Figure 2:**
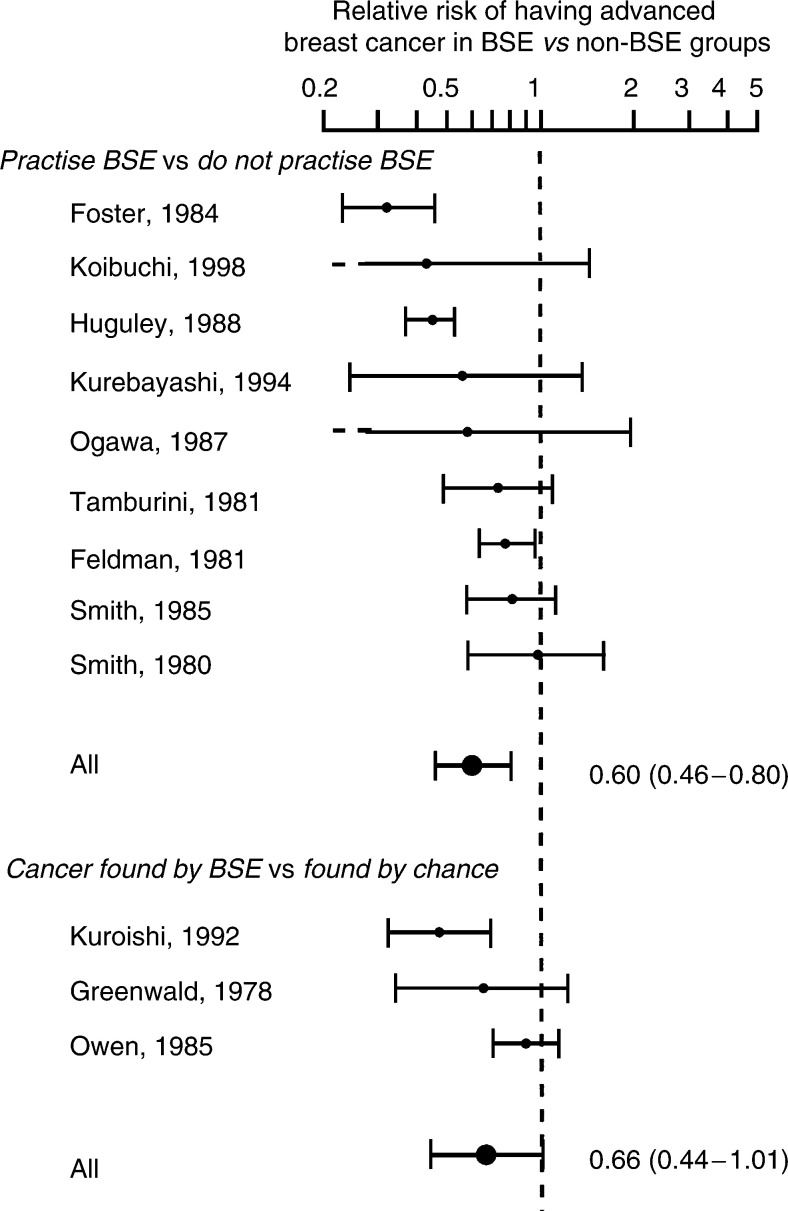
Observational studies of women with breast cancer, comparing the rates of advanced breast cancer between the BSE and non-BSE groups. A test for heterogeneity between the studies yielded a *P*-value of <0.001 for those studies based on women who practise BSE and a *P*-value of 0.051 for those based on finding cancer by BSE.

shows the relative risk of having advanced breast cancer in women who practise BSE compared to those who did not, among all women newly diagnosed with breast cancer. There is a 40% reduction in the risk (relative risk 0.60, 95% CI 0.46–0.80, *P*<0.001). Although there was evidence of hetero-geneity (*P*<0.001), all the studies reported a reduction in risk. In women who found their cancer during an examination, there was a 34% reduction in risk (relative risk 0.66, 95% CI 0.44–1.01, *P*=0.06).

The results from the cohort and case–control studies of women with and without breast cancer according to BSE practice are shown in Table 2

**Table 2:** Observational studies of women with and without breast cancer; number of deaths and relative risk of dying from breast cancer in women who practise BSE compared to those who do not

		**Women who practise BSE regularly**		**Women who do not practise BSE**		
**Study (first author), country**	**Age of women (years)**	**No. of breast cancer deaths^a^**	**No. of women without breast cancer**	**No. of breast cancer deaths^a^**	**No. of women without breast cancer**	**Relative risk or odds ratio of death (95% CI, if available)**
*Cohort studies*						
Gastrin, 1994, Finland	All (⩾20)	95	28 780	—	—	0.71 (0.57–0.87)
	20–49	24	—	—	—	0.64
	⩾50	71	—	—	—	0.74
Holmberg, 1997, USA	All	925	176 677	1375	271 179	1.03 (0.95–1.12)
	⩽39	—	—	—	—	0.95
	40–49	—	—	—	—	1.07
	50–59	—	—	—	—	1.03
	⩾60	—	—	—	—	1.02
						
*Case–control studies*						
Muscat, 1992, USA	All (unspec.)	251	430	184	457	1.45 (1.15–1.83)
Newcomb, 1991, USA	20–80	168	344	41	89	1.06 (0.70–1.60)
Harvey, 1997, Canada	All (40+)	97	1095	121	1091	0.79 (0.59–1.04)

a Or women with advanced breast cancer (Muscat, 1992; Newcomb, 1991). Dashes indicate that the data were not available from the published paper.

. The two cohort studies show inconsistent results; one indicates a statistically significant 29% reduction in the risk of death associated with BSE practice (relative risk 0.71, 95% CI 0.57–0.87) and the other shows no effect at all (relative risk 1.03, 95% CI 0.95–1.12). The pooled estimate is not statistically significant (relative risk 0.87, 95% CI 0.62–1.23, *P*=0.42). None of the case–control studies found statistically significant effects with only one suggesting a benefit (Harvey *et al*, 1997). The results for two of the case–control studies were not materially altered after adjustment for mammography use (Newcomb *et al*, 1991; Muscat and Huncharek, 1992).

Table 3

**Table 3:** Clinical trials of BSE; the number of biopsies, breast cancer cases and deaths and the relative risk of dying from breast cancer in women who practise BSE compared to those who do not

		**BSE training**				**No BSE training**				
**Study**	**Age of women (years)**	**Biopsies**	**Breast cancers**	**Deaths**	**Number randomised**	**Biopsies**	**Breast cancers**	**Deaths**	**Number randomised**	**Relative risk of death(95% CI)**
*Nonrandomised*										
UK Trial, 1999	All (45–74)	—	—	661	63 373^b^	—	—	1312	127 123^b^	0.99 (0.87–1.12)^c^
(after 16 years)	45–49^a^	—	—	236	—	—	—	511	—	0.94 (0.80–1.12)
	50–54	—	—	159	—	—	—	318	—	0.96 (0.78–1.18)
	55–59	—	—	189	—	—	—	388	—	0.98 (0.81–1.19)
	60–64	—	—	165	—	—	—	318	—	0.99 (0.81–1.22)
										
*Randomised*										
China	30–69									
(after 5 years)		1788	331	25	133 375	945	322	25	133 665	1.00 (0.58–1.74)
(after 10 years)		3627	857	135	132 979	2398	890	131	133 085	1.03 (0.81–1.31)
										
Russia	40–64									
(after 5 years)		662	190	—	60 221	467	192	—	60 089	—
(after 9 years)		1094	449	99	57 712	757	406	97	64 759	1.15 (0.87–1.52)
(after 13 years)		1138	493	157	57 712	797	446	164	64 759	1.07 (0.86–1.34)

a Includes women from additional cohorts. Dashes indicate that the data were not available from the published paper. Publications: China (Thomas, 1997, 2002) and Russia (Semiglazov, 1992, 1996, 1999).

b For the UK trial, this is the number of women invited to attend BSE training or the number that were not (i.e. in the comparison centres).

c Age adjusted.

provides the main results from the trials of teaching BSE and Figure 3

**Figure 3:**
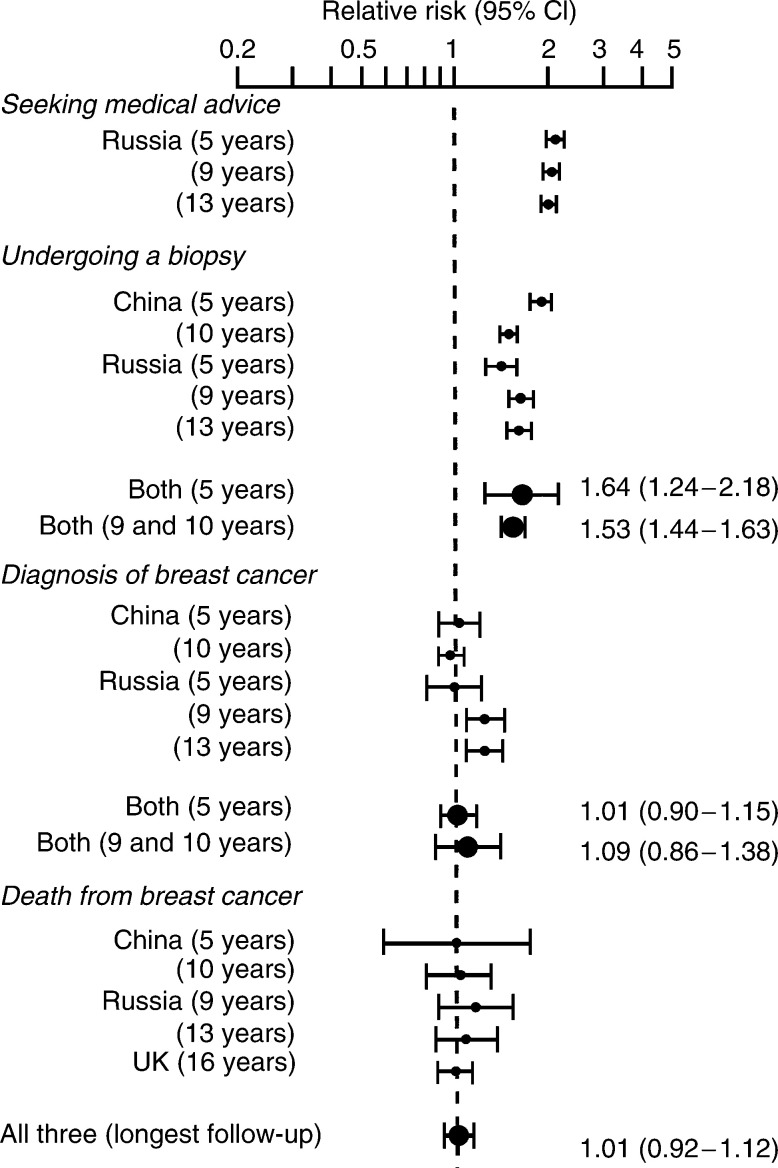
Trials of BSE training. The rates for specified outcomes are compared between women invited for BSE training and those who were not. A test for heterogeneity between the trials yielded a *P*-value of 0.94 in relation to the results on mortality.

shows the relative risks and the pooled estimates for the main outcomes. The nonrandomised trial in the UK showed no effect overall (relative risk 0.99) even after 16 years of follow-up, although there was a difference between the two BSE centres; one showing a reduction in mortality (relative risk 0.79) and the other not (relative risk 1.09), which cannot readily be explained.

In the Russian trial, twice as many women in the BSE group sought medical advice compared to the non-BSE group (Figure 2), and this was consistent throughout the course of the trial (BSE *vs* non-BSE groups: 5.6 *vs* 2.8% at 5 years, 7.2 *vs* 3.5% at 9 years and 7.5 *vs* 3.8% at 13 years). After 10 years, the two trials (Russia and China) show that overall there were 53% more biopsies in women who were taught BSE compared to those who were not, this was highly statistically significant (relative risk of having a biopsy 1.53, 95% CI 1.44–1.63, *P*<0.001). The trials also suggest that at 5 years, women taught BSE were no more likely to be diagnosed with breast cancer than those not taught BSE (relative risk 1.01). After a longer follow-up (9–10 years), there is an indication from the Russian trial that more cancers were found in the BSE group (24% more women diagnosed with breast cancer), but this was not found in the trial from China.

The risk of dying from breast cancer was remarkably consistent between the three trials and over the different follow-up periods. There was no evidence of an advantage in the BSE group after any length of follow-up. The pooled relative risk was 1.01 with narrow 95% confidence limits (0.92–1.12, *P*=0.79); there was no evidence of heterogeneity (*P*=0.94). The results were not materially different if the nonrandomised trial from the UK was excluded, pooled relative risk 1.05 (95% CI 0.90–1.24, *P*=0.54).

There was little evidence that the effect of BSE varied between women in different age groups (Table 1 and Table 2).

## DISCUSSION

### Women who practise BSE

Only observational studies of women with breast cancer who were asked about their history of regular BSE practice consistently found a difference in breast cancer mortality associated with BSE. The studies are likely to be affected by several biases – publication bias, selection bias, recall bias, lead-time bias and length-biased sampling (there may be a larger proportion of slow-growing cancers diagnosed in women who practise BSE; slow-growing cancers tend to have better prognoses). Several studies have shown that various characteristics that are likely to be associated with dying from breast cancer were also associated with BSE practice, but analyses adjusting for the potential effect of such confounding on mortality were not reported. Women who practised BSE tended to be younger, premenopausal and of a higher socioeconomic status (Smith *et al*, 1980; Feldman *et al*, 1981; Tamburini *et al*, 1981; Huguley *et al*, 1988; Le Geyte *et al*, 1992; Auvinen *et al*, 1996). Much of the reduction in mortality observed in these studies might therefore be explained by a combination of these and other confounding factors as well as the aforementioned biases, rather than a real effect of BSE.

### Women who found their cancer during an examination

No evidence of a reduction in mortality was found in women who reported that they found their cancer during self examination (Figure 1). There was an indication that there was an effect when advanced cancer was used as the outcome measure, but the overall result was not statistically significant (Figure 2).

### Women who are taught BSE

The two randomised trials of mortality are unaffected by bias and both show no effect of BSE on breast cancer mortality, after 5 or 13 years. In the Russian trial, there was an increase in breast cancer diagnoses in women taught BSE after 9 and 13 years, but this was not reflected in a decrease in mortality at either time. Both trials also show that women in the BSE group are much more likely to be referred for a biopsy. At about 10 years, the overall malignant to benign biopsy ratio was 1 : 2.3 in the BSE group and 1 : 1.3 in the non-BSE group, indicating that in women who were taught BSE, there is one extra biopsy in women without cancer for every diagnosed case of breast cancer.

Despite the initial appeal of regular BSE, the evidence shows that it is likely to result in a considerable increase in women without breast cancer who have breast biopsy with its associated anxiety and counselling, but with no benefit. The two randomised and one nonrandomised trials were based on about 580 000 women and 2344 breast cancer deaths; the conclusions are therefore robust. Although the two randomised trials were based on BSE training, the negative results are also, to some extent, applicable to BSE practice since uptake was high and women reported practising BSE regularly (every 2 months in the Russian trial and every 4–5 months in the Chinese trial).

### Breast self-examination as an alternative to mammography

The results presented here on BSE may have an impact on the current debate over the use of mammography screening. Despite clear evidence to the contrary (Wald *et al*, 1993; Nystrom *et al*, 1996; IARC 2002), it has been suggested recently that mammography screening is not effective in reducing mortality for breast cancer (Olsen and Gotzsche, 2000,^2001^). Breast self-examination may be considered to be an alternative. The conclusion that mammography was not worthwhile was based on only one out of the six existing randomised trials of breast-cancer mortality comparing mammography with no screening. The other five trials were rejected on the grounds of perceived differences between the screened and unscreened groups at baseline. When the one trial acceptable to the authors was combined with a trial that compared mammography with clinician examination, the reported relative risk was 1.04 (95% CI 0.84–1.27). As a result, there has been some confusion over whether mass mammography screening should continue. Several groups (Reply to Olsen and Gotzsche, 2000; Miettinen *et al*, 2002) have rejected the claim that mammography is not worthwhile with many valid criticisms of the reported analysis. Taking the evidence from all six trials, the relative risk is 0.76 (95% CI 0.67–0.87) in women aged ⩾50 years (Wald *et al*, 1993); a statistically significant 24% reduction in breast cancer deaths. Mammography screening is recommended to women over the age of 40 years in the US and 50–64 years in the UK. Without it, these women currently have no other means of reducing their chance of dying from breast cancer. Breast self-examination, perhaps the only other method that could be in widespread use, is unlikely to be a worthwhile alternative, even as a method of screening to be used in between mammographic examinations. The evidence presented here shows that it is ineffective in saving lives. Women should, of course, still be aware of changes in their breasts and seek advice if concerned, but being taught BSE and practising it regularly is no more effective at reducing breast cancer mortality than finding the tumour by chance.
